# Lab-On-A-Chip for the Development of Pro-/Anti-Angiogenic Nanomedicines to Treat Brain Diseases

**DOI:** 10.3390/ijms20246126

**Published:** 2019-12-05

**Authors:** Subhathirai Subramaniyan Parimalam, Simona Badilescu, Nahum Sonenberg, Rama Bhat, Muthukumaran Packirisamy

**Affiliations:** 1 Optical-Bio Microsystems Laboratory, Micro-Nano-Bio Integration Center, Department of Mechanical and Industrial Engineering, Concordia University, Montreal, QC H3G 2W1, Canada; simonabadilescu0@gmail.com (S.B.); mpackir@encs.concordia.ca (M.P.); 2 Department of Biochemistry, McGill University, Montreal, QC H3A 1A3, Canada; nahum.sonenberg@mcgill.ca; 3 Department of Mechanical and Industrial Engineering, Concordia University, Montreal, QC H3G 2W1, Canada; rama.bhat@concordia.ca

**Keywords:** lab-on-a-chip, microfluidics, brain angiogenesis, nanomedicines

## Abstract

There is a huge demand for pro-/anti-angiogenic nanomedicines to treat conditions such as ischemic strokes, brain tumors, and neurodegenerative diseases such as Alzheimer’s and Parkinson’s. Nanomedicines are therapeutic particles in the size range of 10–1000 nm, where the drug is encapsulated into nano-capsules or adsorbed onto nano-scaffolds. They have good blood–brain barrier permeability, stability and shelf life, and able to rapidly target different sites in the brain. However, the relationship between the nanomedicines’ physical and chemical properties and its ability to travel across the brain remains incompletely understood. The main challenge is the lack of a reliable drug testing model for brain angiogenesis. Recently, microfluidic platforms (known as “lab-on-a-chip” or LOCs) have been developed to mimic the brain micro-vasculature related events, such as vasculogenesis, angiogenesis, inflammation, etc. The LOCs are able to closely replicate the dynamic conditions of the human brain and could be reliable platforms for drug screening applications. There are still many technical difficulties in establishing uniform and reproducible conditions, mainly due to the extreme complexity of the human brain. In this paper, we review the prospective of LOCs in the development of nanomedicines for brain angiogenesis–related conditions.

## 1. Introduction

The human brain is the primary center for our cognitive activities and is commonly recognized as the most complex organ of the human body. Due to its important role in human survival, the brain is guarded by a boney skull (elastic modulus, 2.4 ± 1.5 GPa) [1] and a robust blood–brain barrier (BBB) (Transendothelial Electrical Resistance (TEER), ~5000 Ω cm^2^) [2]. While excellent for protecting the brain, these natural barriers have also made it difficult to study and treat brain disorders, in particular, diseases that require rapid treatment. More specifically, not much is known about up- or downregulation of angiogenesis, that is, the formation of new blood capillaries via vascular sprouting from the preexisting vasculature. Most drugs that are used for treating extracranial angiogenesis (pro-/anti-angiogenic)-related conditions are ineffective intracranially. Timely delivery of the therapeutic molecules across the BBB in an angiogenesis-related condition is therefore needed.

Numerous nanomedicines (NMs) have been investigated to treat angiogenesis-related conditions all over the body, and various endothelium-targeting nanosystems have been reported [3]. Recently, NMs have been investigated for their efficacy in treating brain angiogenesis conditions. These have included nano-capsules, such as solid lipid nanoparticles (SLNs) [4,5,6,7,8], liposomes [9,10,11,12,13,14,15,16], exosomes [17], and others [18], as well as nano-scaffolds made of chitosan [19], polymers [20,21], inorganic nanoparticles (NPs) [22,23,24], etc. (see Table 1). Only a few are FDA approved—namely, lomustine-liposomes and carboplatin-liposomes—and the survival benefits are very limited [25]. While NMs aid in overcoming the anatomical barriers, most of the NM’s clinical viability is still unclear due to the lack of suitable pre-clinical models for drug screening.

The disproportionate and expensive failures during the clinical transformation of the angiogenic brain drugs, have led to the divestment in brain drug development [26]. It is difficult to reproduce the mechanistic aspects of the brain’s vasculature, such as dynamic flow, shear stress, perfusability, and cellular tension, in a static petri dish. Moreover, the sedimentation of NMs in a static culture, and the lack of precise spatial and temporal control renders the petri dish deficient in studying NMs [27]. Also, the limitation in imaging neovascular growth and manipulation of the chemical and biological parameters has made the animal models less desirable. Further, the genetic variation and the difficulty in developing global standard protocols for animal studies [28] have made researchers look for alternatives.

Recently, vascularized microfluidic LOCs have revolutionized the in vitro tissue and organ models, and resulted in the development of vascularized models of, retina [29], skin [30], hair [31], bone [32], thyroid [33], heart [34], lungs, [35], kidney [36], pancreas [37], liver [38], and intestine [39], respectively. A vascularized body-on-chip, linking eight different organs, including the brain and a BBB, transforming the future drug screening technology, was also reported [40]. The human-like perfusability, dynamic microenvironment, and the ability to carry out robust, rapid and reproducible assays in a controlled operational condition, with high throughput screening readouts, have made the above devices a super-tool to screen angiogenic drugs [41]. Some of the above devices were already evaluated for drug screening [32,34,36,38,40], and a few studied the effect of NPs [35,41] and NMs [39].

Microfluidic technology has also been used to model brain vasculogenesis/angiogenesis [42,43,44,45,46], BBB [2,42,47,48,49,50,51,52,53], brain tissues [54,55], and brain angiogenesis-related cellular events such as inflammation [47,50], cell migration [56], cell-cell interactions [57], etc., see Table 2. In addition, specific conditions, involving pathological-angiogenesis, such as brain tumors [46,54,55,56,58], ischemic strokes [59], and neurodegenerative disorders including Alzheimer’s disease (AD) [60], Parkinson’s disease (PD) [61], and Huntington’s disease (HD) [62], could also be created on-chip.

In addition to this, LOCs have been developed for studying the biocompatibility, cellular uptake and transport of NMs [2,27], many of them focusing on brain angiogenesis [2,45,63]. Further, LOCs are also used to synthesis NMs [64]. The prospective of LOCs in the development of nanomedicines for brain angiogenesis-related conditions are discussed here.

### The Brain Vasculature: General Background

The brain microvasculature channels blood to various regions of the brain—a key activity for orchestrating brain function and repair regulated by angiogenesis. A 600 km of small capillaries (7 μm in diameter) supply the brain with constant nourishment, resulting in a brain vessel density (BVD) that is so high that each neuron has its own blood vessel, positioned at a distance of 20 μm from each other. The BVD varies among the different brain regions (e.g., the BVD of cerebral cortex, ~1311 mm^−3^ and white mater, ~222 mm^−3^ [65]) and between healthy and diseased tissues [66]. The brain cells become hypoxic when they are further away from the vessels than 100 to 200 μm, inducing angiogenesis.

**Figure 1:**
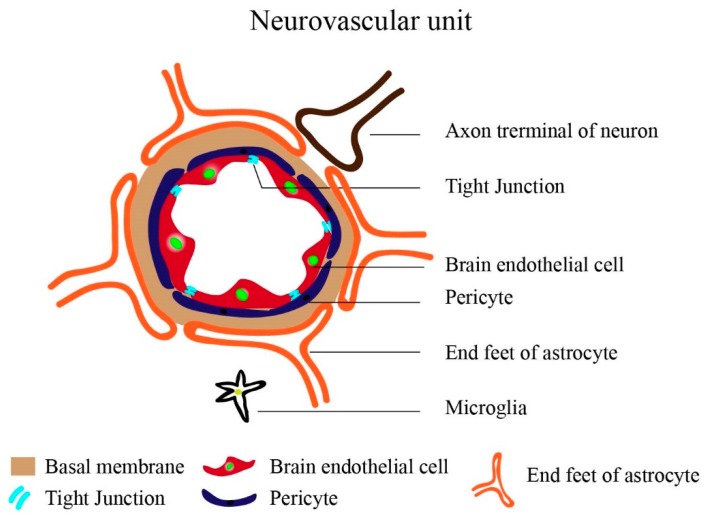
The schematics of neurovascular unit (NUV). The NUV comprises of the brain endothelial cell, pericyte, astrocyte (end feet), neuron, microglia, and the basal membrane.

The brain vasculature is made up of highly specialized, continuous, non-fenestrated brain microvascular endothelial cells (BMECs), (200 nm thick) that are held together by the tight junction (TJ) proteins (zonula occludens (ZOs), cingulin, and 7H6 phosphoprotein, heterotrimeric G-proteins, occludin, claudins, and Junctional adhesion molecules (JAMs)), sealing the interendothelial space. The BMECs have a negative surface charge due to the sulphated proteoglycan, with glycosaminoglycan (anionic) sidechain and are apicobasally polarized, the basal side faces the vessel lumen and is in contact with the blood [67,68]. The apical side faces the brain tissues and are closely associated with pericytes [69], astrocytes [70], immune cells-microglia and myocytes [71] and neurons [72]. Due to this association, a 30–40 nm thick basal membrane (BM) is developed; the above cells along with the BM constitute the neurovascular unit (NVU) (Figure 1).

This NVU hosts the BBB that maintains a stable ionic microenvironment for the propagation of the neuronal action potentials. The TJs proteins render the BBB impermeable, blocking the diffusion of polar solutes, and maintains the integrity, permeability, and the high electric resistance of the BBB. Any oral/intravenous drug should cross the BBB in order to reach the targeted site, and, for this reason, BMEC-targeting NMs are been developed. There are two ways to cross over the BBB: the energy dependent transcellular pathways (receptor-mediated transcytosis), right through the cytosol of BMECs, and the passive paracellular pathways, via the space in between adjacent BMECs. The transcellular pathways are regulated by the molecular receptor-transporters and efflux pumps. In contrast, the paracellular space is guarded by the TJs proteins and requires to be temporarily disrupted to enable drug transport. The junction proteins coordinate with the transmembrane proteins and cytoskeletons to transport materials across the BBB [73].

The BMECs are not fixed in a post-mitotic state and, therefore, respond to various pathological signals and modulate angiogenesis [74]. Subsequently, angiogenesis is tightly regulated by the micro-environment and steered by the pro-vascular endothelial growth factor (VEGF), basic fibroblast growth factor, transforming growth factor beta inflammatory factor and tumor necrosis factor, and hypoxia (hypoxia-inducible transcription) factor, and anti-angiogenic factors, namely, thrombospondin, angiostatin, endostatin, and dopamine agonists [75]. The BBB is closely affiliated with the angiogenesis process in maintaining brain hemostasis. For example, the VEGF that enhances angiogenesis, also increases the permeability of BBB during both physiological [76] and pathological conditions [77]. The characteristics of brain drugs greatly depend on the properties of the BBB.

## 2. Nano-Drug Delivery for Treating Brain Diseases

Bolus delivery of drugs is not effective in achieving the therapeutic concentrations in the targeted brain regions, mainly due to the presence of BBB and intracranial pressure. In order to cross the hydrophobic and highly resistant BBB, the material should be cationic and lipophilic (lipophilicity in the range of 1.5–2.7) and have a hydrodynamic radius in the range of ~200 nm, a molecular weight < 400 Da, and a cumulative number of hydrogen bonds < 8–10. It can be very challenging to develop a drug molecule with the above characteristics/components, without compromising its therapeutic properties. One of the potential alternatives to overcome this challenge is to engineer NMs that take advantage of the properties of BBB and their transcellular routes [78].

NMs are defined as therapeutic particles in the size range of 10–1000 nm, which have unique pharmacokinetic behaviors attributed to their “size effects,” such as increased reactivity due to increased surface area and increase in cellular uptake due to their small size [79,80] and charge [11,81]. Eventually, in order to access the transcellular routes at the BBB, the size, surface chemistry and the charge are found to be critical factors. The cut-off size for the clathrin- and calveolin- mediated endocytosis are 200 and 80 nm in diameter, respectively. But, various studies have demonstrated the uptake of NMs larger than 200 nm (see Table 1). The surface chemistry of the NM’ is such that the circulation time is prolonged and/or complements the receptors on the BMECs.

The surface charge of the NM, is measured in vitro, as the Zeta potential (electrokinetic potential of a particle in a colloidal system). In theory, the BMEC membrane is negatively charged and NMs with a positive surface charge are prone to adsorb strongly to the cell membrane, resulting in a higher cellular intake. However, the experimental results show that liposomes with negative charge tend to exhibited higher cellular uptake at the BBB when compared with the positive and neutral liposomes, respectively [11]. It is believed that the negative/neutral NMs, due to their poor protein adsorption, have a longer circulation time, resulting in a greater probability to be adsorbed to BBB. Although, there is no “golden rule” regarding the characteristic features of the NMs, a right combination of particle size, surface chemistry, and charge is required to cross the BBB.

The NMs can be engineered using various materials and crafted into different shapes and sizes to take advantage of the transcellular and paracellular routes. More importantly, they can be specifically functionalized to target particular pathways and sites on the BMECs. They are designed to have greater BBB permeability [2,6,9,11,17,19], stability [5,9], half-life [11,18,82,83] and shelf-life [5], high-site specific targeting [10,14,84], and a controlled load-release of drugs [18,19,21,84,85].

Medications are either encapsulated into a nano-capsules, or else adsorbed onto nano-scaffolds, which allows them to be delivered across the BBB. During transcellular transport, NMs adsorb on to the apical side of the BMECs (charge dependent [11]), imbibed into the BMEC via endocytosis pathways clathrin-dependent/independent and caveolae mediated pathways. The drug is, either released into the intracellular space of BMECs, or transported across into the basal lamina and released into the brain extracellular matrix, depending on their destined site.

Nano-capsules such as SLN, liposomes and exosomes are most commonly used as carriers. They are highly stable and allow the manipulation of the drug release-kinetics. These carriers can be functionalized with specific peptides and proteins, to exploit the endocytosis pathways and also be targeted to specific regions of brain. Simply by encapsulating the drugs into a nano-capsule, the drug was able to cross the BBB [6,18]. The SLNs are spherical solid lipid cores, that can encapsulate hydrophilic molecules, the core is made of triglycerides, fatty acids and waxes, and their size ranges from 400 to 1000 nm [6]. They are preferred for their higher drug loading capacity, best production scalability and longer shelf-life (SLNs are stable for 45 days, at 4 °C [5]). The liposomes are hollow capsules made of a phospholipid bilayer that encapsulates both hydrophilic and lipophilic drugs (small, <100, large, >100 nm, multi lamellar, >500 nm), and they are by far the most effective for brain drug delivery. By simply altering the lipid composition, size, and charge, they can be tailored to target a specific cell or tissue. The only drawback is the low stability and poor drug loading efficiency. The exosomes are nothing but naturally occurring liposomes, also made of phospholipids, a sub-group of extracellular vesicles, with a size range from 50 nm to 120 nm. They are highly biocompatible and readily internalized by brain cells [17]. Nano-scaffolds made from cationic polysaccharides/polymers (10–1000 nm) have been used to adsorb the drug and facilitate its transport across the BBB by masking the drug’s steric effect [20,21].

### 2.1. Pro-Angiogenic Nanomedicine for Brain Diseases

The therapeutic upregulation of angiogenesis is required to treat brain vasculature loss that occurs during strokes, ageing, and neurodegenerative diseases [74], such as AD, PD, and HD. [86]. The brain stroke, which is caused by the constriction of brain blood vessels or hemorrhage requires immediate treatment as the therapeutic window is only 3–4.5 h. Restoring blood circulation and controlling vascular degeneration through angiogenesis are the major strategies for the above conditions. Various pro-angiogenic materials have been reported, some of which already are available as NMs such as Simvastatin [11], VEGF [18,87], nitric oxide (NO) donors [21], ZL006 [10], Cyclosporine A (CsA) [12], and microRNAs [14,17] (see Table 1).

A tightly regulated spatial and temporal distribution of the pro-angiogenic drugs is required to rapidly initiate and maintain angiogenic sprouting. Some drugs have dual effect, depending on the concentration. For example, Simvastatin has a pro- and an anti-angiogenic effect at nano- and micro-concentrations, respectively [88]. In addition, the bolus delivery of VEGF, small molecule NO donors, is not desired due to the poor receptor activation, shorter half-life and initial burst release. A VEGF-loaded liposome, functionalized with transferrin [87], was found to have enhanced the neuroprotective and angiogenic effects on an ischemic stroke brain. A nano-capsule loaded with VEGF, is made of acrylamide-based monomers and a bisacryl plasmin-labile peptide that release small, time-dependent doses of VEGF and upregulates angiogenesis [18]. The VEGF delivered via a nano-capsule was found to have superior therapeutic outcomes when compared with the bolus delivery [18]. In another study, angiogenesis was induced by releasing low concentrations of NO, from NO donors with short half-life, such as N-diazeniumdiolate and S-nitrosothiol. They were loaded onto nano-scaffolds made of methoxy poly(ethylene glycol)-b-poly(lactic-co-glycolic acid) (mPEG-PLGA) [21].

### 2.2. Anti-Angiogenic Nanomedicine for Brain Diseases

The pathogenic upregulation of angiogenesis occurs due to genetic alterations in tumor cells and/or by hypoxia and plays a critical role in brain cancer biology. Any tumor cannot grow beyond 1–2 mm without developing new blood vessels. Therefore, blocking tumor-induced angiogenesis is one of the main strategies in treatment. From the perspective of drug delivery, brain tumors have additional barriers caused by the efflux activity of the tumor cells. These barriers are the major challenges when treating brain tumors. Bevacizumab (BVZ) [4,20], Temozolomide, (TMZ) [19], Doxorubicin (DOX) [16] Paclitaxel [8], and Sorafenib (SFN) [15] are some of the anti-angiogenic drugs that have been proven to be effective when transported across the BBB using nano-carriers (see Table 1).

The drug effect of BVZ was found to increase up to 100–200 folds when delivered via SNPs [4]. In another study, BVZ was lipolyzed and encapsulated into PLGA NPs along with trehalose in order to achieve long term stability [20]. Indirubin, a hydrophobic compound to treat glioblastoma multiforme (GBM) due to its anti-tumor and anti-angiogenesis properties, was found to be more effective when delivered via SLN [5]. Similarly, SLN functionalized with apolipoprotein E (ApoE), a suppressor of angiogenesis and cell invasion, was found to show a greater cellular intake when compared to non-functionalized SLNs [7]. In another study, methotrexate (MTX), an anti-angiogenic prodrug-loaded SNPs conjugated with ApoE, were found to have a superior biodistribution in the brain, when compared to bolus delivery of MTX [6]. Further, TMZ, an anti-angiogenic drug used to treat GBM is fast degrading and difficult to direct specified therapeutic dosages to targeted regions. In order to overcome this flaw, a chitosan-based NP, loaded with TMZ and functionalized with chlorotoxin (CTX) was developed to selectively target the GBM cells [19]. The NM was found to have higher stability, the half-life was seven-fold greater than the free TMZ and had a 2–6-fold higher uptake by the GBM cells [19].

## 3. Lab-on-a-Chip—A Model for Angiogenic Brain Diseases

Various factors need to be synergized to model brain angiogenesis in vitro; this involves the coordination of different cell lines (endothelial and the brain cells) [50], the combination of angiogenic factors, and the presence of shear stress and dynamic flow, etc. The conventional monolayer, Transwell, and vessel-like capillary cells cultures grown in a static petri dishes are insufficient for synergizing and completely neglect dynamic flow and shear stress. Hence have proven to be unsuitable for evaluating brain angiogenesis drugs, thus a platform to synergies these factors towards angiogenesis is required. Moreover, the way in which NPs are loaded into well plates was found to alter the cellular adsorption and uptake, respectively [89]. As a result, carefully designed experiments involving NMs are needed. In addition to the above advantages, LOCs also allow the testing of very low drug concentrations in a dose-dependent manner, even in the range of a few nanomoles per liter, which is corresponds to the therapeutic concentration [45]. Further, simultaneous, high throughput screening of different concentration on the same microfluidic platforms is possible [46,49,58]. The possibility of developing precise brain-like vasculature to perform challenging experiments has encouraged a number of researchers to adapt LOCs for their studies, Table 2.

### 3.1. Angiogenic Nanomedicine Screening in LOCs

**Figure 2:**
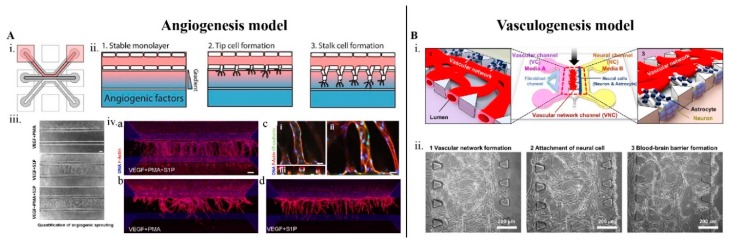
The angiogenesis and vasculogenesis microfluidic model, respectively, for potential angiogenic drug screening application. (**A**) The angiogenesis model [43], i. shows the geometry of the microfluidic device. ii. The device comprises of three parallel microchannel, the endothelial cells are loaded to the top channel (pink), the angiogenic growth factors are added to the bottom channel (blue) and the gradient of the growth factor is generated in the middle channel. 1. Formation of a stable monolayer of the endothelial cells in response to the angiogenic gradient. 2. Cell tip formation followed by 3. The lumen formation. iii. Angiogenic sprouts after four days simulated with different combinations of angiogenic factors, VEGF, phorbol 12-myristate 13-acetate (PMA) and sphingosine-1-phosphate (S1P). iv. (a) Angiogenic sprouts after six days of stimulation with VEGF+PMA+S1P, (b) VEGF+PMA and b) VEGF+S1P, respectively, and (c) close-up of the lumen middle c(i), top c(ii), and cross-section c(iii) and stained against F-actin (red) and nucleus (blue). (**B**) The vasculogenesis model [42], i. The schematics of the in vitro 3D NVU platform comprising of astrocytes, neurons and the endothelial cells. ii. The perfusable vascular network is formed over a three-day period via vasculogenesis. 1. The vascular network formation in the middle channel, 2. Loading of astrocytes and neurons into the right-side channel. 3. The formation BBB within 5–7 days. BBB: blood–brain barrier.

**Figure 3:**
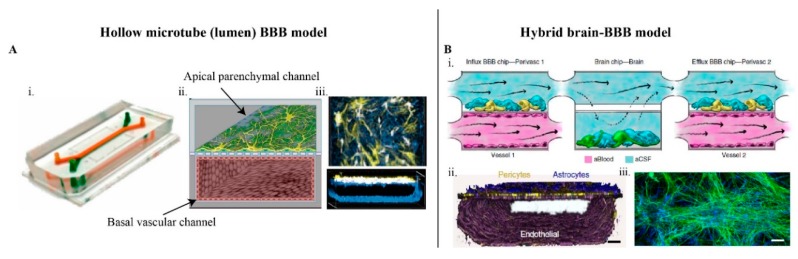
(**A**) Hollow microtube model [2], i. photograph of the PDMS microfluidic device. ii. schematic illustration (center), and immunofluorescence micrographs (right) of the two-channel microfluidic Organ Chip with endothelial cells cultured on all surfaces of the basal vascular channel, and astrocytes and pericytes on the upper surface of the central horizontal membrane in the apical parenchymal channel. iii. At the top pannel, z-stack images of the pericytes (yellow, F-actin staining) and astrocytes (white, glial fibrillary acidic protein (GFAP) staining) in the top channel of the BBB Chip are reconstituted and shown from above; at the bottom panel, a side view of similar stacked images for the lower vascular channel containing endothelial cells (blue, ZO-1 staining). (**B**) (Reprinted with permission from the publisher) i. The hybrid brain-BBB device, displaying the metabolic flux across the BBB and the brain cells, [57], brain endothelial cells (magenta) are cultured on all four walls of the lower vascular compartment and a mixture of brain astrocytes (blue) and pericytes (yellow) in the top compartment of both BBB chips; neuronal cells (green) and astrocytes (blue) are cultured in the lower compartment of the brain chip. Cell culture medium is flowed into the upper perivascular compartment of BBB chip as an artificial cerebrospinal fluid (CSF) (blue), and cell culture medium mimicking blood is flowed separately through the lower vascular compartment. ii. and iii. The reconstruction of the human BBB chip from confocal fluorescence microscopic images. ii. The endothelial cell monolayer stained for VE-Cadherin (purple), and a mixture of pericytes (F-actin, yellow) and astrocytes (GFAP, blue), (scale bar, 75 μm). iii culture of neurons (β-III-tubulin, green) and astrocytes (GFAP, blue) (scale bar, 100 μm). Figure 3B is reproduced with permission from Maoz, B.M.; Herland, A.; Fitzgerald, E.A.; Grevesse, T.; Vidoudez, C.; Pacheco, A.R.; Sheehy, S.P.; Park, T.E.; Dauth, S.; Mannix, R.; et al. A linked organ-on-chip model of the human neurovascular unit reveals the metabolic coupling of endothelial and neuronal cells. *Nat. Biotechnol.* **2018**, *36*, 865–874. [57].

In order to evaluate the pro/anti-angiogenic effects of NMs, their effect on cell migration, angiogenesis, lumen formation, and their ability to target specific sites needs to be studied. In addition, the cellular uptake, transport via the transcellular/paracellular routes and drug-release kinetics, needs to be evaluated. Various device geometries have been reported for brain vasculature models (Figure 2 and Figure 3); parallel channels (PCs), with interconnecting micro-gaps, [42,43,44,45,51,56,90], hollow-microtubes (HTs) [2,47,49,50,52], and others, concentric-ring (CR) channels [54], multi-layered channels [53], hybrid-microchambers [57], and microwells [58]. Each of the above designs has its own advantage in terms of angiogenesis drug screening application.

The PC type LOC (Figure 2), modeling 2D monolayer vascular network, is the most commonly used to model angiogenesis [43,45] (Figure 2A) and vasculogenesis [42,44] (Figure 2B). Usually, five or three microchannels are fabricated (Width, W, 500–1000 um), in the case of vasculogenesis model. The middle channel is loaded with the endothelial cells, and supplied with culture medium through the adjacent channels and the brain cells or fibroblast cells and their respective culture mediums are supplied separately (Figure 2B). In the case of angiogenesis model, the middle channel is supplied with culture medium and the adjacent channels are loaded with endothelial cells (Figure 2A).

The PC type devices enable the study of both the inner and outer surface of the vessels and are best suited for observing angiogenesis. The real-time drug effect on the cell morphology [44,45], cell migration [43,44,45], lumen growth [45], angiogenic sprouting [43,45], vessel anastomosis [43,44], can be observed and quantified in this model. The lumen perfusability is easily achieved in the 2D monolayer and allows for the injection of drugs into the vasculature through the inlet ports. The formation or inhibition of tumor-associated blood vessel, in response to anti-angiogenic drugs can be precisely quantified [45]. In an in vitro brain spheroid, it is not possible to access the lumens to perform perfusability assays. In such cases, the brain spheroids can be introduced form outside into the vascularize device and anastomosed with the preexisting vasculature, thereby enabling lumen access [44,46]. One of the main drawbacks is that it not possible to install electrodes into the PC devices for TEER measurements.

The HT models (Figure 3A) were used to create 3D BBB, with easy-to-access lumens and are useful for studying the apical side of the BBB. The endothelial cells are loaded into the hollow tube, the cells adhere to the tube walls and form a lumen-like structure. In some cases, the brain cells are grown on the outer surface of the tube and were coupled with the endothelial cells via microchannels [47,52], micropores [2] or extracellular matrix [49,50]. The real-time uptake of NMs and their effect on the TJs [47] and transmigration of the neutrophils [47], antibodies [49], and Q-dots functionalized with Angiopep-2 [2], were easily quantified in this model. The BBB permeability during pathological conditions involving neuroinflammation [47,50] and ischemic obstruction were observed in the HT model [47]. A microrod model, i.e., an inside-out geometry of the HT model, demonstrated that BMEC resists elongation in response to both curvature and shear stress [91]. The cell curvature is directly proportionate to the number of adjacent cells around the perimeter and eventually increase the TJs [91], mimicking more closely the in vivo condition.

Apart from the above mentioned advantages, HT models eliminate the artificial paracellular leaks that occurs in Transwells [52], allowing precise measurement of the NMs perfusability [2]. Further, the HT models not only capture the shear stress and the vessel curvature, it is the most convenient model to integrate electrodes for a real-time measurement of TEER without destroying the cells [2,53].

The CR model is advantageous in mimicking tumor angiogenesis conditions, such as radial oxygen gradient on-chips [54], and are suitable for analyzing drugs targeting tumor mediated-angiogenesis. Another recently reported hybrid BBB-brain on-chip device recapitulated brain-blood/CSF flow and the structural hierarchy of the brain tissues (Figure 3B) [57]. The device screened the effect of Methamphetamine, (anti-angiogenic and psychostimulant drug) on the reversible disruption of BBB. The drug uptake, influx and efflux of the drug across the BBB were demonstrated for their potential to screening NMs.

### 3.2. Design and Fabrication of Brain-Angiogenesis LOCs

LOCs are made of optically transparent polymers, namely, polydimethylsiloxane (PDMS), polymethylmethacrylate (PMMA), SU-8, etc., and fabricated by soft lithography from SU-8 patterned molds. The media reservoir, inlets and outlets can be created with a biopsy punch and other fabrication methods. The polymer chip can be bonded to glass coverslips and petri plates using the plasma bond technique and can be sterilized by autoclave and/or UV. Through simple fabrication techniques, electronics can be integrated into the LOC for various applications. For example, electrodes were fabricated to generate an electric field for evaluating barrier functions, such as permeability [51], and TEER [2,53] respectively. Further, the QDs were used to measure brain cell temperature [92]. In addition, stable drug concentration profiles can be easily generated in a microfluidic platform by controlling the volumetric flow rates across multiple-channels [45,58]. Because of these features, non-destructible, real-time, and simultaneous measurement of various factors, such as BBB permeability, TEER, etc., is possible.

### 3.3. Establishing Brain Vasculature on Chips

In order to vascularize the device, the endothelial cells (see Table 2) are co-cultured with one or more cells constituting the NVU. Although, a mono-culture of endothelial cells alone can produce vasculature [42,44,46,50], a more realistic model requires the co-culturing of astrocytes [43,45,47,51] and pericytes [50], or both [2,49,57]. In recent models, neurons [42,57] and tumor cells [46,54] were also included. Direct contact with the above non-endothelial cells, in particular astrocytes and pericytes were found to produce stable BBB, with high TEER and in vivo like permeability, for a longer time period.

Until recently, primary and immortalized cells from both animals and humans, respectively, were used to develop brain angiogenesis models. The recent development of the gene editing technology, such as CRISPR/Cas9, led to the use of Human induced pluripotent stem cells (HiPSC) derived from both healthy and diseased humans [93]. Although it is very challenging to maintain and standardize HiPSC protocols, microfluidics platforms once again have aided in sustaining more complex, dynamic and controlled environment for using HiPSC [2]. The HiPSCs demonstrated superior barrier function and low inflammatory response when compared with the primary cells [2].

In general, it takes around 3–4 days to vascularize a microfluidic device, the endothelial cells are loaded along with the extracellular matrix, and the cells differentiates and polarizes in response to the growth factors (e.g., VEGF) that is supplied by the culture media or released from the co-cultured cell lines. A monolayer or 3D vascular network is formed depending on the device geometry. Following this, vessel-like structure with a lumen is formed and the perfusability is achieved in a few days and the functional BBB develops within the next 5–7 days [42]. The vasculature inside the LOC can be maintained for many weeks and the BBB integrity for up to a full week [2]. The choice of cell lines, composition of the co-cultures, cell loading sequence, culture media flow rate, and mechanical property of the cell matrix, can be easily optimized based on the final applications. Apart from developing the 2D vascular network, 3D brain micro-spheroids (diameter of 100–600 um) were also used to model tumor angiogenesis [44,46,54,55]. The spheroids were grown through one of these techniques, namely, 3D bio-printing [54,55] or the hanging droplet technique [94]. They can also be grown using the micro-wells [58] or 96-well plate and later transferred into the LOC [44]. Recently, a microfluidic platform that can be integrated with a 96-well plate to develop tumor spheroid angiogenesis model was reported [46].

### 3.4. Important Features of Vascularized LOCs Vs. Petri Dish Models

#### 3.4.1. Dynamic Flow and Shear Stress in Brain-Angiogenesis LOCs

Dynamic flow and shear stress play an important role in the cell differentiation, the expression of the membrane proteins, signal pathways and other barrier properties such as TEER. These factors determine the extent to which the brain-like conditions are mimicked on-chip. Physiologically or pathologically relevant expression of protein/peptide receptors involved in the transcellular and paracellular pathways is very important for the clinical analysis of the NMs. The stress promotes glycalyx formation in the endothelial cells, eventually affecting the charge on the membrane, further the non-uniform shear stress affects the endothelial permeability [95]. Therefore, shear stress is an important factor while studying the cellular uptake of NMs. Unlike in the static petri dish culture, dynamic flow and shear stress can be generated on microfluidic platforms. LOC provides precise control over the flow rate and shear stress when integrated with a syringe pump [90]. In an LOC, it is possible to dynamically flow culture media and other fluids at a physiological flow rate and create shear stress on the BMVECs (6 dyne cm^−2^ at 100 μL h^−1^). It is also feasible to recreate blood-like viscosity (3–4 cP) by adding microbeads.

Recently, various studies have reported the significance of these mechanistic factors in mimicking the characteristic features of brain vasculature in LOCs. It was demonstrated that, unlike other endothelial cells, BMEC resists elongation, in response to both curvature and shear stress [91]. Likewise, the TJ proteins were upregulated in a dynamic flow culture, when compared to a static culture [52]. Currently, iPS-BMVECs are used in brain studies as these cells exhibit high levels of TEER (~3000–5000 Ω·cm^2^) within 24–48 h of culture [2]. However, in a static culture, the TEER levels can be maintained only for ~2 days and the junction protein expressions are not high enough, limiting their use in drug screening applications. A transendothelial impedance as high as ~ 25,000 Ohm, was achieved in a dynamic flow LOC and were maintained for a period of one week [2]. In the same study, the expression of P-gp (permeability glycoprotein, efflux transporter) under dynamic flow (100 μL h^−1^), enabled the observation of a 2.7 fold increase in DOX flux [2].

#### 3.4.2. Lumen Perfusability in Brain-Angiogenesis LOCs

It is important that the vasculature is perfusable, in order to mimic the circulation of nutrients and metabolites in the brain, which in turn helps the lumen formation and maintenance [45]. The lumen perfusability was found to increase the neural activity, that was measured by the Ca^2+^ oscillation [44]. While lumen formation can be achieved in petri plate, only LOCs allow direct accesses to them via the chip’s access ports. This enables studying perfusability [96], intra lumen flow [44], drug kinetics, drug translocation across the BBB and their effect on angiogenesis [45,46], in a clinically relevant manner.

The lumen structures with diameters in the range of 1–50 μm can be modeled in an LOC [96], and the intra lumen flow can be generated using a hydrostatic pressure head, simply by connecting the inlet with a larger media reserve [44] and to syringe pumps [50]. The lumens are modeled either by forming a vascular network with innate lumens [42,43,44,46,49] or by recreating the lumen structure, devoid of any vascular network, as described in the HT models [2,47,48,49,50,52]. The perfusability and the integrity of the vasculature were evaluated using microbeads and the dextran, 20 kDa and 70 kDa FITC with Stokes’ radii of 23 and 60 Å, respectively [42,43,44,46,49].

#### 3.4.3. Compartmentalization in Brain-Angiogenesis LOCs

In a conventional petri dish, it is difficult to maintain both endothelial and brain cells simultaneously that require different growth conditions. LOCs enable (simplify) complex system level experiments of multi-culture cell system by providing confined culturing compartments for different cell types, along with their optimal culture media, and tunable brain-like interfaces between them to regulate their interaction. These tunable brain-like interfaces are realized by discontinuous micropillars [42,43,44,45,56], micropores [2,52,53], or extracellular matrix [49] and can be coupled or uncoupled as required to control the migration of molecules and cells between different channels. Direct [42,44,50] or indirect [45,46,52,53] contact between the endothelial cells and the brain cells is achieved through the microstructures.

Better control over the experiments by channelizing and enhancing cell signals, provide a clear understanding about the distinct contribution of various cellular factors. The cell migration [45,46,56], angiogenic sprouting, in response to chemical clues/gradients [42,43,44,46], respectively, and other complex brain cell interactions were modeled in LOCs. For example, the astrocyte migration towards the vascular network, resulting in the enhancement of BBB [42], similarly migration of the endothelial cells in response to growth factor gradient, were observed [43,45,46]. Recently, a hybrid device, displaying the metabolic flux across the BBB and the brain cells were reported [57]. Two BBB chips were connected on either side of a brain chip and for the first time and a BBB influx/efflux through the artificial CSF was demonstrated (Figure 3B). This device replicated the in vivo CSF and blood, flow on-chip. In the brain chip part of the device, by restricting the active flow only to the upper compartment, the flow velocity on the neurons in the lower compartment was zero, similar to the in vivo condition.

### 3.5. LOCs for the Synthesis of NMs

Another major challenge for NMs is the difficulty in reproducing particle synthesis, especially in the case of complex NPs [97]. Microfluidic platforms allow the control of various critical synthesis parameters (temperature, flow rate, reaction rate, etc.) and are used to rapidly synthesize therapeutic NPs in the desired size range, shape and composition [64,98]. Recently, LOCs for producing monodispersed liposomes [99], chitosan NPs [100], protein NPs [101], lipid NPs [102] and other organic NPs [103], some of them with higher EE, were reported [99,102,103].

## 4. Conclusions

In the last decade, various LOCs modeling brain vasculature has been reported, both for fundamental studies and drug screening applications. The LOCs are now beyond the proof of concept stage, but there is still not enough work in this field to allow for the development of standardized devices. There are many technical difficulties in establishing uniform and reproducible conditions, mainly due to the extreme complexity of the human brain. There are very few commercially available LOCs that can be easily adapted for screening NMs [52]. Nonetheless, collaborative efforts from multiple disciplines of science and engineering are necessary to develop LOC protocols for testing NMs for brain angiogenesis conditions.

Brain angiogenesis is a vital process for brain function and development. The pathological upregulation or downregulation of brain angiogenesis is life-threatening and requires rapid treatment. The knowledge of the regulating mechanisms in the brain angiogenesis at the cellular and molecular level, at both physiological and pathological levels, remains insufficient, mainly due to the lack of brain models that closely mimic brain micro-environments. This creates further challenges for the drug development and screening processes.

In order to treat brain angiogenic conditions, various strategies have been developed, the most promising of which is the use of NMs. NMs are efficient in crossing the BBB, which is one of the biggest obstacles in delivering brain drugs. Further, NMs allows programmed and controlled release of drugs in the brain, thereby generating therapeutic concentrations in targeted regions.

Regardless of the rapid growth in the field of NMs, the transition to clinical and pre-clinical stages is still in an incipient stage. The delay is mainly due to the lack of understanding of how the NMs will interact with the complex human brain. So far, only a few NMs have been examined in clinical trials, with even fewer actually reaching the market. There are a couple of shortcomings regarding the use of NPs to treat brain-related conditions. For example, NPs have been found to induce amyloidogenicity [104], and, for this reason, it is important in the future to characterize their complex dynamic surface properties.

## Figures and Tables

**Table 1:** List of nanomedicines (NMs) for brain angiogenesis–related conditions.

S.No.	NM Formulation *	Particle Size (nm)	Zeta Potential (mV)	PDI	EE%	LC%	Disease Model	Ref.
**Pro-angiogenic NMs**								
1	microRNA-210-Exosome-*c(RGDyK) peptide* ^a^	~140	-	-	-	-	Ischemic brain, A	2019, [17]
2	NO donor-Nanocapsule-*PEG-PLGA*	200	1.59 ± 0.254	1.48–1.53	70 ± 4	-	Non-specific, P	2018, [21]
3	PirB-Liposome	100	-	0.201 ± 0.034	-	-	Ischemic stroke, A	2018, [13]
4	CsA-Liposome	81.5 ± 0.75	−37.1	0.056 ± 0.02	78.8 ± 0.59	-	Ischemic neuroinflamation, A	2017, [12]
5	ZL006- Liposome-*T7-SHp* ^b^	96.24 ± 1.13	−3.237 ± 0.206	0.157 ± 0.015	79.12 ± 3.44	9.37 ± 0.48	Ischemic stroke, P	2016, [10]
6	Simvastatin-Liposome	151.85	−1.01	0.15	64.37 ± 7.55	-	Ischemic stroke, A	2016, [11]
7	VEGF-Nanocapsule- *peptide* ^c^	22 ± 3	-	-	-	-	Non-specific, P & A	2016, [18]
8	L-Peptide- Liposome	127.6 ± 48.0	-	-	-	62.1	Ischemic stroke, A	2015 [9]
**Anti-angiogenic NMs**								
1	Indirubin-SLN	118	−16.3 ± 8.11	0.104	99.73	0.054	GBM, P	2019, [5]
2	BVZ-Nano-scaffold-*PLGA, trehalose*	208–238	−6.37	0.09–0.14	84.7 ± 0.3	-	Non-specific, P	2018, [20]
3	SFN-nano-capsule	54 ± 1	−7.8 ± 0.6	0.15 ± 0.01	>90	-	GBM, P & A	2018, [15]
4	MTX-SLN-*ApoE*	338.0±10.0	−7.18 ± 1.92	~0.287	89	1.4	GBM, P & A	2017, [6]
5	SLN-*ApoE, Palmitate*	174 ± 10.3	−11.46	0.156 ± 0.092	-	-	Non-specific, P	2017, [7]
6	Palcitexel-SLN	80–90	−17.4 to −24.8	0.19 ± 0.02	~88	5.18 ± 0.14	GBM, P & A	2016, [8]
7	TMZ-Nano-capsule-*CTX*	~67.2	−1.8 ± 4.3	-	-	4.9 ± 0.5	GBM, P & A	2015, [19]
8	microRNA-21-Liposome-*CTX*	190	Neutral	< 0.3	85–95	-	GBM, P & A	2015, [14]
9	BVZ-SLN-stearic acid	515.6 ± 113.6	-	0.191	29.8 ± 4.4	30.0 ± 5.0	GBM, P	2015 [4]
10	Dox-Liposome	111 ± 5.3	-	-	-	-	GBM, A	2013, [16]
**Others**								
1	QD-*Angiopep-2*	20	-	-	-	-	LOC	2018, [2]
2	Cerium oxide NP	1–10	-	-	-	-	P	2017, [22]
3	Gallium NP	5–7	-	-	-	-	A	2017 [23]

Petri dish model-P, animal model-A, polydispersity index-PDI, encapsulation efficacy-EE, loading capacity-LC. * NM formulation: drug-type of NM (carrier/scaffold/micelles, etc.)-the substance used to functionalize the NM, in italic. ^a^ cyclo (Arg-Gly-Asp-D-Tyr-Lys) peptide, ^b^ stroke homing peptide, ^c^ acrylamide-based monomers and bisacryl plasmin-labile peptide.

**Table 2:** List of labs-on-a-chip (LOCs) modeling brain vasculature.

S.No.	Model	Drug Screening	Dynamic Flow	Lumen perfusability	Vessel Dia.	Endothelial Cells	Brain Cells	Other Cells	TEER	Ref.
1	GBM (spheroid)-angiogenesis (PC)	BVZ, Sunitinib, Cetuximab	Y	Y	-	HUVEC	U87MG	hLF	NA	2019, [46]
2	BBB (HT)	-	Y	Y	W = 200 μm, H = 100 μm	hCMEC/D3	hA	-	NA	2019, [52]
3	GBM-angiogenesis (CR)	TMZ	Static	N	-	HUVEC	U87MG	-	NA	2019, [54]
4	BBB (HT)	Dox, Cetuximab, Q-dot-Angiopep-2	Y	Y	W = 1000 μm, H = 200 μm	iPS-BMVEC	hP, hA	-	Impedance, ~25,000 Ω	2018, [2]
5	Angiogenesis 3D (PC)	-	Y	Y	D = 25 μm	HUVEC	-	-	NA	2018, [43]
6	BBB (HT)	Antibody MEM-189	Y	Y	NA	TY10	hBPCT*, hA	-	NA	2018, [49]
7	GBM spheroid (Microwell)	TMZ, BEV	Static	N	NA	-	GBM cell*	-	NA	2018, [58]
8	Vasculogenesis (PC)	-	Static	Y	-	HUVEC	E17-brain cells	hLF	NA	2017, [42]
9	Vasculogenesis (spheroid) (PC)	-	Y	Y	D = 60 μm	HUVEC, iPS-EC	hNSC	-	NA	2017, [44]
10	Hybrid-Brain (others)	Methamphetamine	Y	N	NA	hBMVEC	hP, HIP-009, hA	-	NA	2017. [57]
11	BBB (HT)	-	Y	Y	D = 600–800 μm	hBMVEC	hP, hA	-	NA	2016, [50]
12	Angiogenesis (PC)	Bortezomib	Y	Y	-	HUVEC		-	NA	2015, [45]
13	BBB (HT)	-	Y	Y	H = 50 μm	RBE4		-	NA	2015, [47]
14	BBB (others)	Mannitol	Y	N	NA	b.End3	C8D1A	-	Resistance, 250 Ω cm^2^	2012, [53]

Yes-Y, Parallel channel-PC, Hollow microtube channel-HT, Co-centric rings-CR, not applicable-NA, Cell line description: HUVEC-Human Umbilical Vein Endothelial Cell (primary), hCMEC-Human Cerebral Microvascular Endothelial Cell (immortal), iPS-BMVEC-induced pluripotent stem cell-derived human brain microvascular endothelial cell, TY10-human spinal cord microvascular endothelial cell (immortal), iPS-EC- induced pluripotent stem cell-derived human endothelial cell, hBMVEC- Human brain microvascular endothelial cells (primary), RBE4-rat brain endothelial cell (immortal), b.End3-mice brain endothelial cell (immortal), U87MG-human glioblastoma cell (immortal), hA- Human Astrocyte (primary), hP-Human brain Pericyte (primary), hBPCT-human brain pericytes (immortal), hNSC- human neuronal stem cell, HIP-009-Human Hippocampal Neural Stem, C8D1A-mice astrocyte cell (immortal), and hLF-human Lung fibroblast (primary). * Patient derived cells.

## Abbreviations

BBB	Blood brain barrier
LOC	Lab-on-a-chip
TEER	Transendothelial Electrical Resistance
NMs	Nanomedicines
SLNs	Solid lipid nanoparticles
NPs	Nanoparticles
CSF	Cerebrospinal fluid
AD	Alzheimer’s disease
PD	Parkinson’s disease
HD	Huntington’s disease
BVD	Brain vessel density
BMECs	Brain microvascular endothelial cells
VEGF	Vascular endothelial growth factor
NO	Nitric oxide
CsA	Cyclosporine A
mPEG-PLGA	Methoxy poly(ethylene glycol)-b-poly(lactic-co-glycolic acid)
BVZ	Bevacizumab
TMZ	Temozolomide
DOX	Doxorubicin
SFN	Sorafenib
GBM	Glioblastoma multiforme
ApoE	Apolipoprotein E
MTX	Methotrexate
CTX	Chlorotoxin
PC	Parallel channel
HT	hollow-microtube
CR	Concentric-ring
PMA	Phorbol 12-myristate 13-acetate
S1P	Sphingosine-1-phosphate
GFAP	Glial fibrillary acidic protein
PDMS	Polydimethylsiloxane
P-gp	Permeability glycoprotein
HUVEC	Human Umbilical Vein Endothelial Cell
hCMEC	Human Cerebral Microvascular Endothelial Cell
iPS-BMVEC	Induced pluripotent stem cell-derived human brain microvascular endothelial cell
hBMVEC	Human brain microvascular endothelial cells
RBE4	Rat brain endothelial cell 4
b.End3	Mice brain endothelial cell
hA	Human Astrocyte
hP	Human brain Pericyte
hNSC	Human neuronal stem cell
HIP-009	Human Hippocampal Neural Stem
C8D1A	Mice astrocyte cell
LF	Human Lung fibroblast

## References

[1] Boruah S., Henderson K., Subit D., Salzar R.S., Shender B.S., Paskoff G. Response of human skull bone to dynamic compressive loading. Proceedings of the 2013 IRCOBI Conference Proceedings—International Research Council on the Biomechanics of Injury.

[2] Park T.-E., Mustafaoglu N., Herland A., Hasselkus R.M., Mannix R., FitzGerald E.A., Prantil-Baun R., Watters A., Henry O., Benz M. (2018). Hypoxia-enhanced Blood-Brain Barrier Chip recapitulates human barrier function, drug penetration, and antibody shuttling properties. bioRxiv.

[3] Flühmann B., Ntai I., Borchard G., Simoens S., Mühlebach S. (2019). Nanomedicines: The magic bullets reaching their target?. Eur. J. Pharm. Sci..

[4] Battaglia L., Gallarate M., Peira E., Chirio D., Solazzi I., Giordano S.M.A., Gigliotti C.L., Riganti C., Dianzani C. (2015). Bevacizumab loaded solid lipid nanoparticles prepared by the coacervation technique: Preliminary in vitro studies. Nanotechnology.

[5] Rahiminejad A., Dinarvand R., Johari B., Nodooshan S.J., Rashti A., Rismani E., Mahdaviani P., Saltanatpour Z., Rahiminejad S., Raigani M. (2019). Preparation and investigation of indirubin-loaded SLN nanoparticles and their anti-cancer effects on human glioblastoma U87MG cells. Cell Biol. Int..

[6] Battaglia L., Muntoni E., Chirio D., Peira E., Annovazzi L., Schiffer D., Mellai M., Riganti C., Salaroglio I.C., Lanotte M. (2017). Solid lipid nanoparticles by coacervation loaded with a methotrexate prodrug: Preliminary study for glioma treatment. Nanomedicine.

[7] Neves A.R., Queiroz J.F., Lima S.A.C., Reis S. (2017). Apo E-Functionalization of Solid Lipid Nanoparticles Enhances Brain Drug Delivery: Uptake Mechanism and Transport Pathways. Bioconjug. Chem..

[8] Banerjee I., De K., Mukherjee D., Dey G., Chattopadhyay S., Mukherjee M., Mandal M., Bandyopadhyay A.K., Gupta A., Ganguly S. (2016). Paclitaxel-loaded solid lipid nanoparticles modified with Tyr-3-octreotide for enhanced anti-angiogenic and anti-glioma therapy. Acta Biomater..

[9] Hwang H., Jeong H.S., Oh P.S., Na K.S., Kwon J., Kim J., Lim S., Sohn M.H., Jeong H.J. (2015). Improving cerebral blood flow through liposomal delivery of angiogenic peptides: Potential of ^18^F-fdg pet imaging in ischemic stroke treatment. J. Nucl. Med..

[10] Zhao Y., Jiang Y., Lv W., Wang Z., Lv L., Wang B., Liu X., Liu Y., Hu Q., Sun W. (2016). Dual targeted nanocarrier for brain ischemic stroke treatment. J. Control. Release.

[11] Campos-Martorell M., Cano-Sarabia M., Simats A., Hernández-Guillamon M., Rosell A., Maspoch D., Montaner J. (2016). Charge effect of a liposomal delivery system encapsulating simvastatin to treat experimental ischemic stroke in rats. Int. J. Nanomed..

[12] Partoazar A., Nasoohi S., Rezayat S.M., Gilani K., Mehr S.E., Amani A., Rahimi N., Dehpour A.R. (2017). Nanoliposome containing cyclosporine A reduced neuroinflammation responses and improved neurological activities in cerebral ischemia/reperfusion in rat. Fundam. Clin. Pharmacol..

[13] Wang J., Zhang Y., Xia J., Cai T., Du J., Chen J., Li P., Shen Y., Zhang A., Fu B. (2018). Neuronal PirB upregulated in cerebral ischemia acts as an attractive theranostic target for ischemic stroke. J. Am. Heart Assoc..

[14] Costa P.M., Cardoso A.L., Custódia C., Cunha P., de Almeida L.P., de Lima M.C.P. (2015). MiRNA-21 silencing mediated by tumor-targeted nanoparticles combined with sunitinib: A new multimodal gene therapy approach for glioblastoma. J. Control. Release.

[15] Clavreul A., Roger E., Pourbaghi-Masouleh M., Lemaire L., Tétaud C., Menei P. (2018). Development and characterization of sorafenib-loaded lipid nanocapsules for the treatment of glioblastoma. Drug Deliv..

[16] Bredlau A.L., Motamarry A., Chen C., McCrackin M.A., Helke K., Armeson K.E., Bynum K., Broome A.M., Haemmerich D. (2018). Localized delivery of therapeutic doxorubicin dose across the canine blood-brain barrier with hyperthermia and temperature sensitive liposomes. Drug Deliv..

[17] Zhang H., Wu J., Wu J., Fan Q., Zhou J., Wu J., Liu S., Zang J., Ye J., Xiao M. (2019). Exosome-mediated targeted delivery of miR-210 for angiogenic therapy after cerebral ischemia in mice. J. Nanobiotechnol..

[18] Zhu S., Segura T. (2016). Cell-Demanded VEGF Release via Nanocapsules Elicits Different Receptor Activation Dynamics and Enhanced Angiogenesis. Ann. Biomed. Eng..

[19] Fang C., Wang K., Stephen Z.R., Mu Q., Kievit F.M., Chiu D.T., Press O.W., Zhang M. (2015). Temozolomide nanoparticles for targeted glioblastoma therapy. ACS Appl. Mater. Interfaces.

[20] Sousa F., Cruz A., Pinto I.M., Sarmento B. (2018). Nanoparticles provide long-term stability of bevacizumab preserving its antiangiogenic activity. Acta Biomater..

[21] Yang C., Hwang H.H., Jeong S., Seo D., Jeong Y., Lee D.Y., Lee K. (2018). Inducing angiogenesis with the controlled release of nitric oxide from biodegradable and biocompatible copolymeric nanoparticles. Int. J. Nanomed..

[22] Sack-Zschauer M., Bader S., Brenneisen P. (2017). Cerium Oxide Nanoparticles as Novel Tool in Glioma Treatment: An In vitro Study. J. Nanomed. Nanotechnol..

[23] Moustafa E.M., Mohamed M.A., Thabet N.M. (2017). Gallium nanoparticle-mediated reduction of brain specific serine protease-4 in an experimental metastatic cancer model. Asian Pacific J. Cancer Prev..

[24] Cheng R., Huang W., Huang L., Yang B., Mao L., Jin K., Zhuge Q., Zhao Y. (2014). Acceleration of tissue plasminogen activator-mediated thrombolysis by magnetically powered nanomotors. ACS Nano.

[25] Gilert A., MacHluf M. (2010). Nano to micro delivery systems: Targeting angiogenesis in brain tumors. J. Angiogenes. Res..

[26] Gribkoff V.K., Kaczmarek L.K. (2017). The need for new approaches in CNS drug discovery: Why drugs have failed, and what can be done to improve outcomes. Neuropharmacology.

[27] Zhu D., Long Q., Xu Y., Xing J. (2019). Evaluating nanoparticles in preclinical research using microfluidic systems. Micromachines.

[28] Osborne N., Avey M.T., Anestidou L., Ritskes-Hoitinga M., Griffin G. (2018). Improving animal research reporting standards. EMBO Rep..

[29] Achberger K., Probst C., Haderspeck J., Bolz S., Rogal J., Chuchuy J., Nikolova M., Cora V., Antkowiak L., Haq W. (2019). Merging organoid and organ-on-a-chip technology to generate complex multi-layer tissue models in a human retina-on-a-chip platform. Elife.

[30] Sriram G., Alberti M., Dancik Y., Wu B., Wu R., Feng Z., Ramasamy S., Bigliardi P.L., Bigliardi-Qi M., Wang Z. (2018). Full-thickness human skin-on-chip with enhanced epidermal morphogenesis and barrier function. Mater. Today.

[31] Abaci H.E., Coffman A., Doucet Y., Chen J., Jacków J., Wang E., Guo Z., Shin J.U., Jahoda C.A., Christiano A.M. (2018). Tissue engineering of human hair follicles using a biomimetic developmental approach. Nat. Commun..

[32] Marturano-Kruik A., Nava M.M., Yeager K., Chramiec A., Hao L., Robinson S., Guo E., Raimondi M.T., Vunjak-Novakovic G. (2018). Human bone perivascular niche-on-a-chip for studying metastatic colonization. Proc. Natl. Acad. Sci. USA.

[33] Bulanova E.A., Koudan E.V., Degosserie J., Heymans C., Pereira F.D.A.S., Parfenov V.A., Sun Y., Wang Q., Akhmedova S.A., Sviridova I.K. (2017). Bioprinting of a functional vascularized mouse thyroid gland construct. Biofabrication.

[34] Jastrzebska E., Tomecka E., Jesion I. (2016). Heart-on-a-chip based on stem cell biology. Biosens. Bioelectron..

[35] Huh D., Matthews B.D., Mammoto A., Montoya-Zavala M., Yuan Hsin H., Ingber D.E. (2010). Reconstituting organ-level lung functions on a chip. Science.

[36] Homan K.A., Gupta N., Kroll K.T., Kolesky D.B., Skylar-Scott M., Miyoshi T., Mau D., Valerius M.T., Ferrante T., Bonventre J.V. (2019). Flow-enhanced vascularization and maturation of kidney organoids in vitro. Nat. Methods.

[37] Nguyen D.-H.T., Lee E., Alimperti S., Norgard R.J., Wong A., Lee J.J.-K., Eyckmans J., Stanger B.Z., Chen C.S. (2019). A biomimetic pancreatic cancer on-chip reveals endothelial ablation via ALK7 signaling. Sci. Adv..

[38] Lee J.B., Park J.S., Shin Y.M., Lee D.H., Yoon J.K., Kim D.H., Ko U.H., Kim Y.T., Bae S.H., Sung H.J. (2019). Implantable Vascularized Liver Chip for Cross-Validation of Disease Treatment with Animal Model. Adv. Funct. Mater..

[39] Carvalho M.R., Barata D., Teixeira L.M., Giselbrecht S., Reis R.L., Oliveira J.M., Truckenmüller R., Habibovic P. (2019). Colorectal tumor-on-a-chip system: A 3D tool for precision onco-nanomedicine. Sci. Adv..

[40] Novak R., Ingram M., Clauson S., Das D., Delahanty A., Herland A., Maoz B.M., Jeanty S.S.F., Somayaji M.R., Burt M. (2019). A robotic platform for fluidically-linked human body-on-chips experimentation. bioRxiv.

[41] Phan D.T.T., Wang X., Craver B.M., Sobrino A., Zhao D., Chen J.C., Lee L.Y.N., George S.C., Lee A.P., Hughes C.C.W. (2017). A vascularized and perfused organ-on-a-chip platform for large-scale drug screening applications. Lab Chip.

[42] Bang S., Lee S.R., Ko J., Son K., Tahk D., Ahn J., Im C., Jeon N.L. (2017). A Low Permeability Microfluidic Blood-Brain Barrier Platform with Direct Contact between Perfusable Vascular Network and Astrocytes. Sci. Rep..

[43] Van Duinen V., Zhu D., Ramakers C., van Zonneveld A.J., Vulto P., Hankemeier T. (2019). Perfused 3D angiogenic sprouting in a high-throughput in vitro platform. Angiogenesis.

[44] Osaki T., Sivathanu V., Kamm R.D. (2018). Engineered 3D vascular and neuronal networks in a microfluidic platform. Sci. Rep..

[45] Kim C., Kasuya J., Jeon J., Chung S., Kamm R.D. (2015). A quantitative microfluidic angiogenesis screen for studying anti-angiogenic therapeutic drugs. Lab Chip.

[46] Ko J., Ahn J., Kim S., Lee Y., Lee J., Park D., Jeon N.L. (2019). Tumor spheroid-on-a-chip: A standardized microfluidic culture platform for investigating tumor angiogenesis. Lab Chip.

[47] Cho H., Seo J.H., Wong K.H.K., Terasaki Y., Park J., Bong K., Arai K., Lo E.H., Irimia D. (2015). Three-dimensional blood-brain barrier model for in vitro studies of neurovascular pathology. Sci. Rep..

[48] Vatine G.D., Barrile R., Workman M.J., Sances S., Barriga B.K., Rahnama M., Barthakur S., Kasendra M., Lucchesi C., Kerns J. (2019). Human iPSC-Derived Blood-Brain Barrier Chips Enable Disease Modeling and Personalized Medicine Applications. Cell Stem Cell.

[49] Wevers N.R., Kasi D.G., Gray T., Wilschut K.J., Smith B., Vught R., Shimizu F., Sano Y., Kanda T., Marsh G. (2018). A perfused human blood-brain barrier on-a-chip for high-throughput assessment of barrier function and antibody transport. Fluids Barriers CNS.

[50] Herland A., Van Der Meer A.D., FitzGerald E.A., Park T.E., Sleeboom J.J.F., Ingber D.E. (2016). Distinct contributions of astrocytes and pericytes to neuroinflammation identified in a 3D human blood-brain barrier on a chip. PLoS ONE.

[51] Bonakdar M., Graybill P.M., Davalos R.V. (2017). A microfluidic model of the blood-brain barrier to study permeabilization by pulsed electric fields. RSC Adv..

[52] Brown T.D., Nowak M., Bayles A.V., Prabhakarpandian B., Karande P., Lahann J., Helgeson M.E., Mitragotri S. (2019). A microfluidic model of human brain (μHuB) for assessment of blood brain barrier. Bioeng. Transl. Med..

[53] Booth R., Kim H. (2012). Characterization of a microfluidic in vitro model of the blood-brain barrier (μBBB). Lab Chip.

[54] Yi H.G., Jeong Y.H., Kim Y., Choi Y.J., Moon H.E., Park S.H., Kang K.S., Bae M., Jang J., Youn H. (2019). A bioprinted human-glioblastoma-on-a-chip for the identification of patient-specific responses to chemoradiotherapy. Nat. Biomed. Eng..

[55] O’Cearbhaill E. (2019). 3D bioprinting chips away at glioblastomal resistance. Sci. Transl. Med..

[56] Ayuso J.M., Monge R., Martínez-González A., Virumbrales-Muñoz M., Llamazares G.A., Berganzo J., Hernández-Laín A., Santolaria J., Doblaré M., Hubert C. (2017). Glioblastoma on a microfluidic chip: Generating pseudopalisades and enhancing aggressiveness through blood vessel obstruction events. Neuro. Oncol..

[57] Maoz B.M., Herland A., Fitzgerald E.A., Grevesse T., Vidoudez C., Pacheco A.R., Sheehy S.P., Park T.E., Dauth S., Mannix R. (2018). A linked organ-on-chip model of the human neurovascular unit reveals the metabolic coupling of endothelial and neuronal cells. Nat. Biotechnol..

[58] Akay M., Hite J., Avci N.G., Fan Y., Akay Y., Lu G., Zhu J.J. (2018). Drug Screening of Human GBM Spheroids in Brain Cancer Chip. Sci. Rep..

[59] Mauleon G., Fall C.P., Eddington D.T. (2012). Precise spatial and temporal control of oxygen within in vitro brain slices via microfluidic gas channels. PLoS ONE.

[60] Jorfi M., D’Avanzo C., Tanzi R.E., Kim D.Y., Irimia D. (2018). Human Neurospheroid Arrays for In Vitro Studies of Alzheimer’s Disease. Sci. Rep..

[61] Fernandes J.T.S., Chutna O., Chu V., Conde J.P., Outeiro T.F. (2016). A novel microfluidic cell co-culture platform for the study of the molecular mechanisms of Parkinson’s disease and other synucleinopathies. Front. Neurosci..

[62] Virlogeux A., Moutaux E., Christaller W., Genoux A., Bruyère J., Fino E., Charlot B., Cazorla M., Saudou F. (2018). Reconstituting Corticostriatal Network on-a-Chip Reveals the Contribution of the Presynaptic Compartment to Huntington’s Disease. Cell Rep..

[63] Hajal C., Campisi M., Mattu C., Chiono V., Kamm R.D. (2018). In vitro models of molecular and nano-particle transport across the blood-brain barrier. Biomicrofluidics.

[64] Valencia P.M., Farokhzad O.C., Karnik R., Langer R. (2012). Microfluidic technologies for accelerating the clinical translation of nanoparticles. Nat. Nanotechnol..

[65] Kubíková T., Kochová P., Tomášek P., Witter K., Tonar Z. (2018). Numerical and length densities of microvessels in the human brain: Correlation with preferential orientation of microvessels in the cerebral cortex, subcortical grey matter and white matter, pons and cerebellum. J. Chem. Neuroanat..

[66] Bohn K.A., Adkins C.E., Mittapalli R.K., Terrell-Hall T.B., Mohammad A.S., Shah N., Dolan E.L., Nounou M.I., Lockman P.R. (2016). Semi-automated rapid quantification of brain vessel density utilizing fluorescent microscopy. J. Neurosci. Methods.

[67] Ando Y., Okada H., Takemura G., Suzuki K., Takada C., Tomita H., Zaikokuji R., Hotta Y., Miyazaki N., Yano H. (2018). Brain-Specific Ultrastructure of Capillary Endothelial Glycocalyx and Its Possible Contribution for Blood Brain Barrier. Sci. Rep..

[68] Worzfeld T., Schwaninger M. (2016). Apicobasal polarity of brain endothelial cells. J. Cereb. Blood Flow Metab..

[69] Brown L.S., Foster C.G., Courtney J.M., King N.E., Howells D.W., Sutherland B.A. (2019). Pericytes and neurovascular function in the healthy and diseased brain. Front. Cell. Neurosci..

[70] Michinaga S., Koyama Y. (2019). Dual roles of astrocyte-derived factors in regulation of blood-brain barrier function after brain damage. Int. J. Mol. Sci..

[71] Zhao X., Eyo U.B., Murugan M., Wu L.J. (2018). Microglial interactions with the neurovascular system in physiology and pathology. Dev. Neurobiol..

[72] Andreone B.J., Lacoste B., Gu C. (2015). Neuronal and Vascular Interactions. Annu. Rev. Neurosci..

[73] Berndt P., Winkler L., Cording J., Breitkreuz-Korff O., Rex A., Dithmer S., Rausch V., Blasig R., Richter M., Sporbert A. (2019). Tight junction proteins at the blood–brain barrier: Far more than claudin-5. Cell. Mol. Life Sci..

[74] Sweeney M.D., Kisler K., Montagne A., Toga A.W., Zlokovic B.V. (2018). The role of brain vasculature in neurodegenerative disorders. Nat. Neurosci..

[75] Szade A., Grochot-Przeczek A., Florczyk U., Jozkowicz A., Dulak J. (2015). Cellular and molecular mechanisms of inflammation-induced angiogenesis. IUBMB Life.

[76] Jiang S., Xia R., Jiang Y., Wang L., Gao F. (2014). Vascular endothelial growth factors enhance the permeability of the mouse blood-brain barrier. PLoS ONE.

[77] Janelidze S., Hertze J., Nägga K., Nilsson K., Nilsson C., Wennström M., van Westen D., Blennow K., Zetterberg H., Hansson O. (2017). Increased blood-brain barrier permeability is associated with dementia and diabetes but not amyloid pathology or APOE genotype. Neurobiol. Aging.

[78] Ceña V., Játiva P. (2018). Nanoparticle crossing of blood-brain barrier: A road to new therapeutic approaches to central nervous system diseases. Nanomedicine.

[79] Shilo M., Sharon A., Baranes K., Motiei M., Lellouche J.P.M., Popovtzer R. (2015). The effect of nanoparticle size on the probability to cross the blood-brain barrier: An in-vitro endothelial cell model. J. Nanobiotechnol..

[80] Trickler W.J., Lantz-Mcpeak S.M., Robinson B.L., Paule M.G., Slikker W., Biris A.S., Schlager J.J., Hussain S.M., Kanungo J., Gonzalez C. (2014). Porcine brain microvessel endothelial cells show pro-inflammatory response to the size and composition of metallic nanoparticles. Drug Metab. Rev..

[81] Lockman P.R., Koziara J.M., Mumper R.J., Allen D. (2004). Nanoparticle surface charges alter blood-brain barrier integrity and permeability. J. Drug Target..

[82] Zhou Y., Peng Z., Seven E.S., Leblanc R.M. (2018). Crossing the blood-brain barrier with nanoparticles. J. Control. Release.

[83] Zhao M., Chang J., Fu X., Liang C., Liang S., Yan R., Li A. (2012). Nano-sized cationic polymeric magnetic liposomes significantly improves drug delivery to the brain in rats. J. Drug Target..

[84] Wang C., Zhu J., Ma J., Yang Y., Cui X. (2019). Functionalized *Bletilla striata* polysaccharide micelles for targeted intracellular delivery of Doxorubicin: In vitro and in vivo evaluation. Int. J. Pharm..

[85] Karim R., Palazzo C., Evrard B., Piel G. (2016). Nanocarriers for the treatment of glioblastoma multiforme: Current state-of-the-art. J. Control. Release.

[86] Hohman T.J., Bell S.P., Jefferson A.L. (2015). The role of vascular endothelial growth factor in neurodegeneration and cognitive decline: Exploring interactions with biomarkers of Alzheimer disease. JAMA Neurol..

[87] Zhao H., Bao X.J., Wang R.Z., Li G.L., Gao J., Ma S.H., Wei J.J., Feng M., Zhao Y.J., Ma W.B. (2011). Postacute ischemia vascular endothelial growth factor transfer by transferrin-targeted liposomes attenuates ischemic brain injury after experimental stroke in rats. Hum. Gene Ther..

[88] Dulak J., Jozkowicz A. (2005). Anti-Angiogenic and Anti-Inflammatory Effects of Statins: Relevance to Anti-Cancer Therapy. Curr. Cancer Drug Targets.

[89] Moore T.L., Urban D.A., Rodriguez-Lorenzo L., Milosevic A., Crippa F., Spuch-Calvar M., Balog S., Rothen-Rutishauser B., Lattuada M., Petri-Fink A. (2019). Nanoparticle administration method in cell culture alters particle-cell interaction. Sci. Rep..

[90] DeStefano J.G., Xu Z.S., Williams A.J., Yimam N., Searson P.C. (2017). Effect of shear stress on iPSC-derived human brain microvascular endothelial cells (dhBMECs). Fluids Barriers CNS.

[91] Ye M., Sanchez H.M., Hultz M., Yang Z., Bogorad M., Wong A.D., Searson P.C. (2014). Brain microvascular endothelial cells resist elongation due to curvature and shear stress. Sci. Rep..

[92] Tanimoto R., Hiraiwa T., Nakai Y., Shindo Y., Oka K., Hiroi N., Funahashi A. (2016). Detection of Temperature Difference in Neuronal Cells. Sci. Rep..

[93] Cochrane A., Albers H.J., Passier R., Mummery C.L., van den Berg A., Orlova V.V., van der Meer A.D. (2018). Advanced in vitro models of vascular biology: Human induced pluripotent stem cells and organ-on-chip technology. Adv. Drug Deliv. Rev..

[94] Nzou G., Wicks R.T., Wicks E.E., Seale S.A., Sane C.H., Chen A., Murphy S.V., Jackson J.D., Atala A.J. (2018). Human cortex spheroid with a functional blood brain barrier for high-throughput neurotoxicity screening and disease modeling. Sci. Rep..

[95] Cicha I. (2016). Strategies to enhance nanoparticle-endothelial interactions under flow. J. Cell. Biotechnol..

[96] Nashimoto Y., Hayashi T., Kunita I., Nakamasu A., Torisawa Y.S., Nakayama M., Takigawa-Imamura H., Kotera H., Nishiyama K., Miura T. (2017). Integrating perfusable vascular networks with a three-dimensional tissue in a microfluidic device. Integr. Biol..

[97] Baer D.R. (2018). The Chameleon Effect: Characterization Challenges Due to the Variability of Nanoparticles and Their Surfaces. Front. Chem..

[98] Badilescu S., Packirisamy M. (2012). Microfluidics-nano-integration for synthesis and sensing. Polymers.

[99] Deshpande S., Dekker C. (2018). On-chip microfluidic production of cell-sized liposomes. Nat. Protoc..

[100] Pessoa A.C.S.N., Sipoli C.C., De La Torre L.G. (2017). Effects of diffusion and mixing pattern on microfluidic-assisted synthesis of chitosan/ATP nanoparticles. Lab Chip.

[101] Van Ballegooie C., Man A., Andreu I., Gates B.D., Yapp D. (2019). Using a microfluidics system to reproducibly synthesize protein nanoparticles: Factors contributing to size, homogeneity, and stability. Processes.

[102] Kimura N., Maeki M., Sato Y., Note Y., Ishida A., Tani H., Harashima H., Tokeshi M. (2018). Development of the iLiNP Device: Fine Tuning the Lipid Nanoparticle Size within 10 nm for Drug Delivery. ACS Omega.

[103] Capretto L., Carugo D., Mazzitelli S., Nastruzzi C., Zhang X. (2013). Microfluidic and lab-on-a-chip preparation routes for organic nanoparticles and vesicular systems for nanomedicine applications. Adv. Drug Deliv. Rev..

[104] Wang B., Pilkington E.H., Sun Y., Davis T.P.T., Ke P.C., Ding F. (2017). Modulating protein amyloid aggregation with nanomaterials. Environ. Sci. Nano.

